# Supplementary material for: Do-calculus enables estimation of causal effects in partially observed biomolecular pathways
Source: Bioinformatics. 2022 Jun 27;38(Suppl 1):i350–8. doi: 10.1093/bioinformatics/btac251 (PMC9235495; doi:10.1093/bioinformatics/btac251)
Supplement: btac251_Supplementary_Data [file btac251_supplementary_data.pdf]

Bioinformatics
doi.10.1093/bioinformatics/xxxxxx
Advance Access Publication Date: 2 April 2015
Applications Note
OXFORD

Systems Biology and Networks

Supplementary materials : Do-calculus enables estimation of causal effects in partially observed biomolecular pathways

Abstract

In the appendix we provide the proof for motivating example 2. We also provide links to the network motifs for case studies 1,2,5, and 6.

1 Motivating example 2 : Proof

We provide the proof in a multivariate situation where $\mathbf{U}$ and $\mathbf{X}$ are multivariate and Z and Y are single variables. The same as in Example 2, assume a linear Gaussian relationship between the variables as follows: $\mathbf{U} := N_{\mathbf{U}}$; $\mathbf{X} := \mathbf{U}\theta_{\mathbf{UX}} + N_{\mathbf{X}}$; $Z := \mathbf{X}\theta_{\mathbf{X}Z} + N_Z$; $Y := Z\theta_{ZY} + \mathbf{U}\theta_{\mathbf{U}Y} + N_Y$. The dimensions of U, and X are $N \times L$ and $N \times J$ respectively where L is the total number of latent variables, J is the total number of causes and N is the total number of data points. The dimensions of μ_U, $\theta_{\mathbf{UX}}$,$\theta_{\mathbf{X}Z}$, θ_{ZY}, and $\theta_{\mathbf{U}Y}$ are $1 \times L$, $L \times J$, $J \times 1$, 1×1, and $L \times 1$ respectively. Furthermore, the same as in Example 2, we assume that the disturbances $\mathbf{N} = \{N_{\mathbf{U}}, N_{\mathbf{X}}, N_Z, N_Y\}$ are multivariate Gaussian where $N_A \sim N(\mu_A, \Sigma_{N_A N_A})$ for $A \in \{\mathbf{U}, \mathbf{X}, Z, Y\}$. Hence,

$$\begin{aligned}
\mathbf{U} &\sim N(\mu, \Sigma_{\mathbf{UU}}) \\
\mathbf{X} &\sim N(\mu\theta_{\mathbf{UX}}, \Sigma_{\mathbf{XX}}) \\
Z &\sim N(\mu\theta_{\mathbf{UX}}\theta_{\mathbf{X}Z}, \Sigma_{ZZ}) \\
Y &\sim N(\mu\theta_{\mathbf{UX}}\theta_{\mathbf{X}Z}\theta_{ZY} + \mu\theta_{\mathbf{U}Y}, \Sigma_{YY}) \\
Y|do(\mathbf{X}=\mathbf{x}) &\sim N(\mathbf{x}\theta_{XZ}\theta_{ZY} + \mu\theta_{\mathbf{U}Y}, \\
&\quad \theta_{ZY}^T\Sigma_{N_Z N_Z}\theta_{ZY} + \theta_{\mathbf{U}Y}^T\Sigma_{\mathbf{UU}}\theta_{\mathbf{U}Y} + \Sigma_{N_Y N_Y}) \qquad (1)
\end{aligned}$$

Let $\Sigma_{\mathbf{UX}ZY}$ be the covariance matrix between all 4 variables,

$$\Sigma_{\mathbf{UX}ZY} = \begin{pmatrix} \Sigma_{\mathbf{UU}} & \Sigma_{\mathbf{UX}} & \Sigma_{\mathbf{U}Z} & \Sigma_{\mathbf{U}Y} \\ \Sigma_{\mathbf{XU}} & \Sigma_{\mathbf{XX}} & \Sigma_{\mathbf{X}Z} & \Sigma_{\mathbf{X}Y} \\ \Sigma_{Z\mathbf{U}} & \Sigma_{Z\mathbf{X}} & \Sigma_{ZZ} & \Sigma_{ZY} \\ \Sigma_{Y\mathbf{U}} & \Sigma_{Y\mathbf{X}} & \Sigma_{YZ} & \Sigma_{YY} \end{pmatrix} \qquad (2)$$

The bottom right 3×3 sub-matrix represents observed variables. Σ_{XX} is $J \times J$, Σ_{XZ} is $J \times 1$, Σ_{XY} is $J \times 1$ and the rest are 1×1. The entries of this sub-matrix are as follows:

$$\begin{aligned}
\Sigma_{XX} &= \theta_{UX}^T\Sigma_{\mathbf{UU}}\theta_{UX} + \Sigma_{N_X N_X} && (3) \\
\Sigma_{XZ} &= \Sigma_{XX}\theta_{XZ} && (4) \\
\Sigma_{XY} &= \Sigma_{XX}\theta_{XZ}\theta_{ZY} + \theta_{UX}^T\Sigma_{\mathbf{UU}}\theta_{UY} && (5) \\
\Sigma_{ZZ} &= \theta_{XZ}^T\Sigma_{XX}\theta_{XZ} + \Sigma_{N_Z N_Z} && (6) \\
\Sigma_{ZY} &= \theta_{XZ}^T\Sigma_{XX}\theta_{XZ}\theta_{ZY} + \theta_{XZ}^T\theta_{UX}^T\Sigma_{\mathbf{UU}}\theta_{UY} && (7) \\
&\quad + \Sigma_{N_Z N_Z}\theta_{ZY} && (8) \\
\Sigma_{YY} &= \theta_{ZY}^T\Sigma_{ZZ}\theta_{ZY} + 2\theta_{UY}^T\Sigma_{\mathbf{UU}}\theta_{UX}\theta_{XZ}\theta_{ZY} && (9) \\
&\quad + \theta_{UY}^T\Sigma_{\mathbf{UU}}\theta_{UY} + \Sigma_{N_Y N_Y} && (10)
\end{aligned}$$

The number of equations in this model exceed the number of unknown parameters. Hence, after training the parameters, there exists an equivalence class of structural equations with parameters $(\theta_{UX1}, \theta_{XZ1}, \theta_{ZY1}, \theta_{UY1}, \Sigma_{\mathbf{UU}1}, \mu_1)$ that are not equal to true parameters $(\theta_{UX}, \theta_{XZ}, \theta_{ZY}, \theta_{UY}, \Sigma_{\mathbf{UU}}, \mu)$ but return the same observable covariance matrices:

Eq. (3): If we replace θ_{UX} and $\Sigma_{\mathbf{UU}}$ with, $\theta_{UX1} := c.\theta_{UX}$ and $\Sigma_{\mathbf{UU}1} = \Sigma_{\mathbf{UU}}/c^2$ respectively, where c is a non-zero constant, then Σ_{XX} stays unchanged.

Eq. (4): θ_{XZ1} should be equal to θ_{XZ}, for Σ_{XZ} to stay unchanged.

Eq. (5): If we set $\theta_{UY1} = c.\theta_{UY}$, and $\theta_{ZY1} = \theta_{ZY}$ then Σ_{XY} stay unchanged.

Eq. (6), 8 and 10: With this setting, Σ_{ZZ}, Σ_{ZY} and Σ_{YY} stay unchanged.

Finally, let's derive the mean of each of the observed variables:

$$E[X] = \mu\theta_{UX};\ E[Z] = \mu\theta_{UX}\theta_{XZ};\ E[Y] = \mu\theta_{UX}\theta_{XZ} \qquad (11)$$

If we set $\mu_1 = \mu/c$, then $E[X]$, $E[Z]$ and $E[Y]$ stay unchanged. If in Eq. (1) we replace the parameters with the estimated parameters $(c.\theta_{UX}, \theta_{XZ}, \theta_{ZY}, c.\theta_{UY}, \Sigma_{\mathbf{UU}}/c^2, \mu/c)$, we obtain the same mean and variance.

In this example, the causal effect of $X = x$ on Y is identifiable and we showed that there is an uncertainty region over μ, , $\Sigma_{\mathbf{UU}}$, θ_{UX}, and

© The Author . Published by Oxford University Press. All rights reserved. For permissions, please e-mail: journals.permissions@oup.com

θ_{UY}, but this uncertainty region does not affect the mean and variance of the distribution of Y under intervention on X ($Y|do(X = x)$). With the same setting and without a mediator, we can't estimate the distribution of $Y|do(X = x)$ without bias.

2 Network motifs for case studies 1,2,5 and 6

To demonstrate the ubiquity of the motif in case studies 1, and 5, we queried the EcoCyc database [1] for all *E. coli* front-door motifs with one or more confounders and one or more causes. The motifs are available at https://ecocyc.org/group?id=biocyc14-15682-3843672784. To demonstrate the ubiquity of the motif in case studies 2 and 6, we queried the EcoCyc database for all *E. coli* Napkin motifs with two or more confounders. The motifs are available at https://ecocyc.org/group?id=biocyc14-15682-3844537443.

References

[1]I. M. Keseler, S. Gama-Castro, A. Mackie, R. Billington, C. Bonavides-Martínez, R. Caspi, A. Kothari, M. Krummenacker, P. E. Midford, L. Muñiz-Rascado, et al. The ecocyc database in 2021. *Frontiers in Microbiology*, page 2098, 2021.
